# The relationship between obesity-related *H19DMR* methylation and *H19* and *IGF2* gene expression on offspring growth and body composition

**DOI:** 10.3389/fnut.2023.1170411

**Published:** 2023-09-21

**Authors:** Perla Pizzi Argentato, Jorge Augusto Petroli Marchesi, Naiara Naiana Dejani, Patrícia Yury Nakandakare, Laísla de França da Silva Teles, Lívia Patrícia Rodrigues Batista, Maria Paula Carvalho Leitão, Liania Alves Luzia, Ester Silveira Ramos, Patricia Helen Rondó

**Affiliations:** ^1^Nutrition Department, School of Public Health, University of São Paulo, São Paulo, Brazil; ^2^Department of Genetics, Ribeirão Preto Medical School, University of São Paulo, São Paulo, Brazil

**Keywords:** maternal obesity, fetal growth, fetal body composition, imprinted gene, DNA methylation, cord blood, maternal decidua, placental villi

## Abstract

**Background and objective:**

Imprinted genes are important for the offspring development. To assess the relationship between obesity-related *H19DMR* methylation and *H19* and *IGF2* gene expression and offspring growth and body composition.

**Methods:**

Thirty-nine overweight/obese and 25 normal weight pregnant women were selected from the “Araraquara Cohort Study” according to their pre-pregnancy BMI. Fetal growth and body composition and newborn growth were assessed, respectively, by ultrasound and anthropometry. The methylation of *H19DMR* in maternal blood, cord blood, maternal decidua and placental villi tissues was evaluated by methylation-sensitive restriction endonuclease qPCR, and *H19* and *IGF2* expression by relative real-time PCR quantification. Multiple linear regression models explored the associations of DNA methylation and gene expression with maternal, fetal, and newborn parameters.

**Results:**

*H19DMR* was less methylated in maternal blood of the overweight/obese group. There were associations of *H19DMR* methylation in cord blood with centiles of fetal biparietal diameter (BPD) and abdominal subcutaneous fat thickness and newborn head circumference (HC); *H19DMR* methylation in maternal decidua with fetal occipitofrontal diameter (OFD), HC, and length; *H19DMR* methylation in placental villi with fetal OFD, HC and abdominal subcutaneous fat thickness and with newborn HC. *H19* expression in maternal decidua was associated with fetal BPD and femur length centiles and in placental villi with fetal OFD and subcutaneous arm fat. *IGF2* expression in maternal decidua was associated with fetal BPD and in placental villi with fetal OFD.

**Conclusion:**

To our knowledge, this is the first study to demonstrate associations of imprinted genes variations at the maternal-fetal interface of the placenta and in cord blood with fetal body composition, supporting the involvement of epigenetic mechanisms in offspring growth and body composition.

## 1. Introduction

The World Health Organization (WHO) alerts to the high prevalence of overweight and obesity worldwide. Obesity is currently one of the leading public health problems, with projections of ~2.3 billion overweight and 700 million obese adults by 2025 (1). Within this scenario, an increase is observed in the incidence of overweight among women of reproductive age (2). Overweight and obesity affect maternal and child health, contributing to the development of maternal diseases such as gestational diabetes and hypertension and exposing fetuses to an unfavorable metabolic environment, which can lead to the development of chronic diseases later in life (3).

The literature shows that imprinted genes are associated with embryonic and placental development, regulating the metabolism and physiology of the offspring (4). Genomic imprinting is an epigenetic process that silences a parental allele, resulting in monoallelic expression. Changes in the expression pattern of these genes can affect fetal growth and weight at birth (5). The placenta exhibits high expression of imprinted genes, and these genes are important for determining the placental phenotype by regulating environmental responses and nutrient transport (6).

Maternal obesity is associated with altered DNA methylation profiles (7). These alterations mainly occur in differentially methylated regions (DMRs), including imprinted genes such as insulin-like growth factor 2 (*IGF2*) (8). The *IGF2* and *H19* genes are two oppositely expressed imprinted genes located adjacent to each other at 11p15.5 that share similar epigenetic transcriptional regulatory mechanisms and play a role in feto-placental development (9).The *H19* differentially methylated region (DMR) is paternally methylated and maternally unmethylated, and regulates the imprinted expression of *H19* and *IGF2* (10).

Animal models involving mice demonstrated that *H19* deletion resulted in increased *IGF2* expression and fetal overgrowth (11), and that *IGF2* deletion resulted in fetal growth restriction (12). In humans, maternal blood *IGF2* methylation was associated with birth weight (13) and *IGF2* expression in the placenta was associated with fetal growth disorders (14). A cohort study in the Netherlands that evaluated fetal growth through serial measurements with ultrasound and weight of infants at 3 and 6 months of life, also found associations of *IGF2* and *H19* DNA methylation with fetal and infant growth (15). Despite efforts to a better understanding of the role of *IGF2* and *H19DMR* in the regulation of offspring growth, their relationship with body composition and different tissues, such as placenta, has not yet been fully clarified.

Thus, in this study, we aimed to investigate the impact of maternal overweight/obesity on: (1) fetal-newborn growth and body composition during two gestational periods and postpartum, (2) *H19DMR* methylation in maternal and umbilical cord blood, maternal decidua and placental villi tissues, (3) the expression of *H19* and *IGF2* genes in the maternal decidua and placental villi tissues, and (4) the relationship between DNA methylation and gene expression in different maternal and fetal tissues with fetal-newborn growth and body composition.

## 2. Subjects and methods

### 2.1. Subjects

This study involved the first 39 overweight/obese and 25 normal weight pregnant women, according to their pre-pregnancy body mass index (BMI), selected in 2017–2018, as part of a large epidemiological prospective study that is still under development and should incorporate 2000 pregnant women—“The Araraquara Cohort Study”. They were followed up during three different periods of pregnancy and at delivery: time 1 (T1), from gestational age ≤ 15 weeks; time 2 (T2), 20–26 weeks; time 3 (T3), 30–36 weeks; and time 4 (T4, at delivery). Pregnant women were eligible for the study after signing the informed consent form approved by the Research Ethics Committee of the School of Public Health, University of São Paulo (protocol number 2.570.576). This study was conducted in compliance with the Helsinki guidelines.

The women were excluded from the study if they had more than 15 weeks of gestation, were under 18 and over 35 years of age, had a pre-pregnancy BMI < 18.5 kg/m^2^ (malnourished), and had a serious mental illness or infectious diseases. Women who miscarried, gave birth to twins, had a stillborn baby or babies with congenital diseases, or failed to attend one appointment during the follow-up were also excluded.

Demographic and socioeconomic (age, race, marital status, and education), lifestyle (smoking and alcohol consumption), obstetric (parity and morbidity), and nutritional (pre-pregnancy BMI and total weight gain) data were obtained by interview and from the medical records.

### 2.2. Anthropometric assessment of the pregnant women

Anthropometric assessment (T1, T2, T3, T4) was performed in an ambulatory room. Maternal weight and body composition were assessed by bioimpedance using the Tanita^®^ MC-180 MA equipment (Tanita^®^, Tokyo, Japan). Pre-pregnancy weight was considered as the weight evaluated up to the 13th week of gestation. Height was measured with a Seca^®^ 206 stadiometer (Seca^®^, Hamburg, Germany). BMI was calculated using weight and height values [weight (kg)/height^2^ (m)] and weight gain in pregnancy was compared to the Institute of Medicine recommendations (16). Trained personnel performed all measurements using standardized procedures.

### 2.3. Fetal growth and body composition

Fetal growth was evaluated by ultrasound at T2 and T3. A trained sonographer performed the assessments with the Siemens ACUSON X300TM ultrasound system, premium edition (Siemens^®^, Mountain View, CA, USA), equipped with abdominal curvilinear transducers (C5-2, C6-3, V7-3). The following biometric measurements were obtained from the fetus: biparietal diameter (BPD, cm), occipitofrontal diameter (OFD, cm), head circumference (HC, cm), abdominal circumference (AC, cm), femur length (FL, cm), humeral length (HL, cm), and length (cm). The fetal growth measurements were classified according to the centiles of the INTERGROWTH-21st (IG-21) charts (17).

The following fetal body composition parameters were assessed: abdominal subcutaneous fat thickness (SCFT, mm); total thigh tissue = total muscle mass + fat (cm^3^); thigh muscle mass = internal area of the subcutaneous tissue of the thigh (cm^3^); subcutaneous thigh fat = total thigh tissue – thigh muscle mass (cm^3^); total arm tissue = arm muscle mass + fat (cm^3^); arm muscle mass = internal area of the subcutaneous tissue of the arm (cm^3^); subcutaneous arm fat = total arm tissue - arm muscle mass (cm^3^).

### 2.4. Anthropometry of the newborns

After delivery (T4), the newborns were weighed on a Soehnle Multina Plus electronic baby scale (Soehnle^®^, Gaildorfer, Germany). Length (cm) was measured with a Seca^®^ 416 infantometer (Seca^®^, Hamburg, Germany) and BMI [birth weight (kg)/length^2^ (m)] was calculated. Newborn AC (cm) and thoracic circumference (TC, cm) were measured with a Seca^®^ 201 flexible tape (Seca^®^, Hamburg, Germany). To ensure accuracy and reproducibility of the measurements, the researchers attended a dedicated training course.

### 2.5. Biological material

A qualified professional collected maternal blood into EDTA VACUETTE^®^ tubes at T3. Cord blood was collected with a sterile slip-tip 20-ml Descarpack^®^ syringe with a 25 × 7 mm needle, transferred without the needle to EDTA VACUETTE^®^ tubes, homogenized manually, and refrigerated. Placenta samples were collected immediately after delivery and the umbilical cord was clamped and removed with scissors for weighing on a Soehnle Multina Plus electronic baby scale (Soehnle^®^, Gaildorfer, Germany). Maternal decidua and placental villi tissues were extracted from the central cotyledon, and the tissues were separated with scissors, forceps, and magnifying glass on a Petri dish. Approximately 0.1 g of the maternal decidua and placental villi tissues were stored separately in sterile Eppendorf^®^ tubes containing 1 ml of RNAlater™ Stabilization Solution (Life Technologies, Vilnius, LT). Approximately 0.3 g of maternal decidua and placental villi tissues were stored separately in sterile Eppendorf tubes containing 1 ml of phosphate-buffered saline (PBS 1X). The tubes were frozen at −80°C for subsequent RNA and DNA extraction.

### 2.6. DNA extraction

Total genomic DNA was extracted from maternal blood, cord blood, maternal decidua and placental villi tissues samples using proteinase K (Invitrogen™, Carlsbad, CA, USA) according to the manufacture's protocol, followed by a modified salting-out method (18). The extracted DNA was quantified in a Nanodrop spectrophotometer (Thermo Fisher Scientific Inc., Santa Clara, CA, USA). Samples with an OD260:OD280 ratio higher than 1.8 and an OD260:OD230 ratio between 1.8 and 2.2 were classified as pure. DNA integrity was evaluated by electrophoresis on 2.0% agarose gel with ethidium bromide and diluted to a concentration of ~50 ng/μL.

### 2.7. Methylation analysis

The methylation-sensitive restriction endonuclease qPCR method was used to determine the level of *H19DMR* methylation. The *H19DMR* sequence obtained from the ENSEMBL database (https://www.ensembl.org/index.html) was used for analysis. The region amplified by the primers comprises a 209 bp sequence located at 4,447 bp after the transcription start site, within the imprinting control region (ICR1) located in the *H19*/*IGF2* locus (Supplementary Figure S1). CpGs islands were predicted with MethPrimer2.0 (http://www.urogene.org/methprimer2/) according to the software's parameters. Primers were designed using the Primer3Plus software, Beacon Designer (http://www.premierbiosoft.com/qOligo/Oligo.jsp?PID=1) for analysis of secondary structures, and Primer-BLAST (https://www.ncbi.nlm.nih.gov/tools/primer-blast/) to confirm specificity. The *H19DMR* primer was designed to flank the restriction site of the methylation-sensitive endonuclease MspI/HpaII (FERMENTAS FastDiges^®^, Waltham, EUA) at one single cleavage site (Supplementary Table S1).

A set of genomic DNA templates were generated from the samples. Each set consisted of three tubes with equal DNA concentrations submitted to the methylation-sensitive enzyme HpaII, the non-methylation-sensitive enzyme MspI, and a non-enzyme digest (mock). The reaction mixture contained 1 U of enzyme per 200 ng of DNA and 1 × CutStart Buffer, in a total reaction volume of 20 μL. For the non-enzyme control, ultrapure water was added instead of enzyme. All prepared samples were incubated at 37°C for 16 h, followed by heat inactivation at 65°C for 5 min.

The qPCR for methylation was performed in a StepOne Real-Time PCR System (Thermo Fisher Scientific, USA) using 5 μL of Power SYBR^®^ Green PCR Master Mix (Applied Biosystems), 2 pmol of each primer (forward and reverse primers; Supplementary Table S1), 2 μL of DNA template, and ultrapure water, in a total volume of 10 μL. The qPCR conditions were: denaturation at 95°C for 10 s, followed by 40 cycles at 95°C for 5 s and annealing and elongation at 60°C for 30 s. Amplification efficiencies were obtained by standard curve analysis and specificities were evaluated by melting curve analysis using the program from 70 to 95°C at 0.3°C/s. The percentage of methylation was determined using the formula (½)^Ctd − Ctnd^, where *Ctd* is the cycle threshold (Ct) of the HpaII-digested DNA and *Ctnd* is the Ct of the non-enzyme control (19).

#### 2.7.1. Restriction site confirmation

Prior to DNA methylation analysis, polymerase chain reaction-restriction fragment length polymorphism (PCR-RFLP) analysis was carried out to confirm the presence of the restriction site of HpaII/MspI (C^∧^CGG) in the populations studied. The PCR for each gene was performed in a volume of 25 μL containing 1 μL of 10 × PCR buffer, 1 mM MgCl_2_, 0.2 mM dNTPs, 1 U of Taq DNA polymerase, 10 pmol of each primer, and 100 ng of genomic DNA. The thermal cycling conditions were 5 min at 94°C, followed by 35 cycles at 94°C for 1 min, 60°C for 40 s, 72°C for 1 min, and a final extension at 72°C for 5 min. The PCR product was digested with 2 U of HpaII in 1 μL of 10 × CutStart buffer and 5 μL of water at 37°C for 16 h, followed by heat inactivation at 65°C for 20 min. The PCR and RFLP products were visualized by 2% agarose gel electrophoresis with ethidium bromide.

### 2.8. mRNA extraction and cDNA synthesis

For extraction of total mRNA, 100 mg of frozen maternal decidua and placental villi samples were macerated in liquid nitrogen and extracted using the PureLink™ RNA Mini Kit (Thermo Fisher Scientific, Carlsbad, CA, USA) following the manufacturer's protocol. Samples with an OD 260:OD280 ratio higher than 1.8 were classified as pure. RNA integrity was confirmed by 1.5% agarose gel electrophoresis for 90 min. First-strand cDNA was synthesized from 4 μg of total RNA using the SuperScript^®^ IV First-Strand Synthesis System (Thermo Fisher Scientific, Vilnius, LT) and oligo dT primers according to manufacturer's recommendations.

### 2.9. Gene expression analysis

For gene expression analysis of the *H19*, tyrosine 3-monooxygenase/tryptophan 5-monooxygenase activation protein zeta (*YWHAZ*) and glyceraldehyde-3-phosphate dehydrogenase (*GAPDH*) genes, primers were designed using the Primer3Plus software, Beacon Designer (http://www.premierbiosoft.com/qOligo/Oligo.jsp?PID=1) for the analysis of secondary structures and Primer-BLAST (https://www.ncbi.nlm.nih.gov/tools/primer-blast/) for the confirmation of exon-exon junction specificity. The *IGF2* primers were described by (8) Supplementary Table S1.

The quantitative PCR (qPCR) assay was performed with the StepOne Real-Time PCR System (Applied Biosystems, USA) using 10 μL of SYBR Green PCR Master Mix (Applied Biosystems), 1 μL (2 pmol) of each primer, and 2 μL of 1:11 diluted cDNA. The amplification conditions for all primers were 95°C for 10 min, followed by 45 cycles at 95°C for 15 s and 65°C for 1 min. A melting curve stage of 70–95°C was added in all qPCR assays to verify their specificity. The reactions were analyzed in duplicate and negative controls were included to detect contamination. Primer efficiency was obtained by linear regression [efficiency = 10 (−1/slope)] and primers with an efficiency of 90–110% were considered for gene expression analysis. The average Ct was collected and the 2-ΔΔCT was calculated (20) for each sample to obtain the fold-change. The *GAPDH* and *YWHAZ* reference genes were used for normalization.

### 2.10. Statistical analysis

Descriptive statistics (mean and standard deviation (SD), frequency, and percentage) were used to summarize the data. The Shapiro-Wilk test was applied to analyze the normality of the data. The *t*-test for independent samples was used for comparison between groups and the effect size (Glass' delta) was calculated. Categorical variables were compared between the two groups of pregnant women by the chi-square test. Spearman's correlation test was used to investigate the correlation of mean DNA methylation and gene expression levels in each tissue with maternal overweight/obesity, fetal biometry and body composition, and newborn anthropometry. Univariate and multiple linear regression models were used to explore the associations of DNA methylation and gene expression levels in each tissue with markers of fetal biometry and body composition and newborn anthropometry. The outcome measures were BPD, OFD, FL, HL, length, HC, AC, SCFT, total thigh tissue, thigh muscle mass, subcutaneous thigh fat, total arm tissue, arm muscle mass, and subcutaneous arm fat of the fetus at T2 and T3, as well as weight, length, HC, PT, and AC of the newborns at T4. The confounding variables included maternal age, pre-pregnancy BMI, gestational weight gain, gestational age, and newborn sex. Statistical significance was established at *p* < 0.05 and analysis was performed using SPSS 18.0 (SPSS, Chicago, IL, USA).

## 3. Results

Table 1 shows the demographic, socioeconomic, and obstetric characteristics of overweight/obese and normal weight pregnant women and their respective newborns. No significant difference was found in age, race, education, parity, or morbidity during pregnancy between groups. Newborns in both groups were similar for gestational age but differed significantly for sex (*p* = 0.021).

**Table 1:** Demographic, socioeconomic, and obstetric characteristics of overweight/obese, and normal weight pregnant women and their newborns.

	**Overweight/obese (*n* = 39)**	**Normal weight (*n* = 25)**	***P***
**Pregnant women**			
Age (years)	26.93 ± 6.47	26.39 ± 6.10	0.795
**Gestational weeks**			
T1	13.00 ± 2.10	12.54 ± 2.16	0.507
T2	23.86 ± 2.00	23.83 ± 1.58	0.353
T3	33.08 ± 1.38	32.77 ± 1.54	0.641
**Race**			
White	22 (56.41%)	14 (56.00%)	0.706
Black	4 (10.27%)	3 (12.00%)	
Yellow^*^	2 (5.13%)	–	
Brown^**^	11 (28.20%)	8 (32.00%)	
**Marital status**			
Single/without partner	6 (15.38%)	1 (4.00%)	0.155
Married/with partner	33 (84.61%)	24 (96.00%)	
**Education**			
Elementary school	19 (48.72%)	8 (32.00%)	0.102
High school degree	19 (48.72%)	13 (52.00%)	
University degree	1 (2.56%)	4 (16.00%)	
**Smoking**			
No	37 (94.87%)	25 (100%)	0.250
Yes	2 (5.13%)	–	
**Alcohol intake**			
No	35 (89.74%)	23 (92.00%)	0.763
Yes	4 (10.27%)	2 (8.00%)	
**Parity**			
0	18 (46.14%)	13 (52.00%)	0.661
1	11 (28.20%)	8 (32.00%)	
2–4	10 (25.64%)	4 (16.00%)	
**Hypertension**			
No	38 (97.44%)	24 (96.00%)	0.747
Yes	1 (2.56%)	1 (4.00%)	
**Diabetes**			
No	35 (89.74%)	24 (96.00%)	0.363
Yes	4 (10.26%)	1 (4.00%)	
**Urinary tract infection**			
No	35 (89.74%)	20 (80.00%)	0.279
Yes	4 (10.26%)	5 (20.00%)	
**Cervicitis**			
No	37 (94.87%)	23 (92.00%)	0.643
Yes	02 (5.13%)	2 (8.00%)	
**Newborns**			
Gestational age in T4	39.49 ± 1.83	39.58 ± 1.07	0.054
**Sex**			
Female	15	17	**0.021**
Male	24	8	

Mean ± SD or number of individuals (percentage). T-test for independent samples or chi square test. T1 = ≤ 15 gestational weeks. T2 = 20–26 weeks. T3 = 30–36 weeks. T4 = delivery.

* Yellow: descendants of people who left East Asia for Brazil.

** Brown: skin colors based on a mixture of skin colors between whites, blacks, and indigenous people.

Table 2 shows that pre-pregnancy weight (*p* = 0.019) and pre-pregnancy BMI (*p* = 0.018), as well as the amount of pre-pregnancy fat mass (*p* = 0.007), were higher in the overweight/obese group compared to the normal weight group. The pregnancy BMI at T2, T3, and T4 remained higher in the overweight/obese group but the total gestational weight gain did not differ significantly between groups. Fetuses of overweight/obese pregnant women showed higher centiles of HC at T2 (*p* = 0.045), BPD (*p* = 0.049) at T3, and OFD at T3 (*p* = 0.049) compared to the normal weight group. Fetal body composition parameters such as SCFT (*p* = 0.021), total arm tissue (*p* = 0.033), arm muscle mass (*p* = 0.046), and arm subcutaneous fat (*p* = 0.029) at T2 were higher in the overweight/obese group compared to the normal weight group. Regarding newborn parameters, HC (*p* = 0.046) was significantly higher in the overweight/obese group compared to the normal weight group. According to the results of the Glass' delta, all maternal, fetal and newborn parameters that showed statistically significant results in the *t*-test, had effect sizes considered very large and/or medium.

**Table 2:** Anthropometry and body composition of overweight/obese and normal weight pregnant women and of their fetuses and newborns.

	**Overweight/obese (*n* = 39)**	**Normal weight (*n* = 25)**	***p***	**Glass' delta**	**Interpretation**
**Pregnant women**					
T1 pre-pregnancy weight (kg)	75.11 ± 11.21	59.47 ± 6.59	**0.019**	**2.37**	**very large**
T1 height (cm)	161.89 ± 7.12	163.55 ± 5.42	0.101	−0.31	small
T1 pre-pregnancy BMI (kg/m^2^)	28.57 ± 3.09	22.17 ± 1.47	**0.018**	**4.35**	**very large**
Pre-pregnancy fat mass (%)	36.16 ± 4.36	28.28 ± 3.83	0.571	**2.06**	**very large**
Pre-pregnancy fat mass (kg)	27.72 ± 7.06	17.05 ± 3.47	**0.007**	**3.07**	**very large**
T2 pregnancy BMI (kg/m^2^)	30.37 ± 3.27	24.13 ± 1.68	**0.000**	**3.71**	**very large**
T3 pregnancy BMI (kg/m^2^)	31.84 ± 3.33	25.88 ± 1.93	**0.000**	**3.09**	**very large**
T4 pregnancy BMI (kg/m^2^)	33.25 ± 3.54	27.57 ± 2.38	**0.000**	**2.39**	**very large**
T4 total gestational weight gain (kg)	12.20 ± 6.14	14.36 ± 4.38	0.154	−0.49	small
**Fetuses**					
**Growth parameters**					
T2 biparietal diameter (cm)	6.00 ± 0.65	5.93 ± 0.60	0.239	0.12	very small
T3 biparietal diameter (cm)	8.61 ± 0.40	8.40 ± 0.40	0.495	**0.52**	**average**
T2 biparietal diameter centiles	46.09 ± 33.50	35.64 ± 28.87	0.395	0.36	small
T3 biparietal diameter centiles	49.89 ± 23.83	37.85 ± 29.70	**0.049**	0.41	small
T2 occipitofrontal diameter (cm)	7.62 ± 0.72	7.43 ± 0.64	0.913	0.30	small
T3 occipitofrontal diameter (cm)	10.61 ± 0.64	10.50 ± 0.63	0.614	0.17	very small
T2 occipitofrontal diameter centiles	48.36 ± 31.12	33.13 ± 27.92	0.999	**0.55**	**average**
T3 occipitofrontal diameter centiles	53.47 ± 33.25	48.09 ± 33.40	**0.049**	0.19	very small
T2 head circumference (cm)	21.18 ± 3.55	21.15 ± 1.84	0.628	0.02	null
T3 head circumference (cm)	30.38 ± 1.35	29.88 ± 1.46	0.597	0.34	small
T2 head circumference centiles	47.49 ± 30.43	33.15 ± 26.45	**0.045**	**0.54**	**average**
T3 head circumference centiles	56.63 ± 29.86	49.21 ± 32.48	0.416	0.23	small
T2 abdominal circumference (cm)	19.14 ± 2.18	18.68 ± 1.72	0.446	0.27	small
T3 abdominal circumference (cm)	29.11 ± 2.16	28.36 ± 2.20	0.541	0.34	small
T2 fetal abdominal circumference centiles	59.21 ± 29.00	47.60 ± 30.34	0.287	0.38	small
T3 fetal abdominal circumference centiles	62.54 ± 25.71	54.49 ± 29.39	0.463	0.27	small
T2 femur length (cm)	4.17 ± 0.51	4.20 ± 0.53	0.863	−0.06	very small
T3 femur length (cm)	6.30 ± 0.37	6.24 ± 0.44	0.426	0.14	small
T2 centiles femur length	56.11 ± 23.05	58.77 ± 30.29	0.596	−0.09	very small
T3 centiles femur length	67.63 ± 27.83	66.83 ± 29.94	0.957	0.03	null
T2 humeral length (cm)	3.89 ± 0.46	3.94 ± 0.46	0.997	−0.11	very small
T3 humeral length (cm)	5.63 ± 0.29	5.55 ± 0.42	0.149	0.19	very small
T2 length (cm)	29.35 ± 3.49	29.38 ± 3.68	0.863	−0.01	very small
T3 length (cm)	44.08 ± 2.62	43.50 ± 3.24	0.339	0.18	very small
**Body composition parameters**					
T2 fetal weight (g)	658.77 ± 190.00	640.88 ± 171.39	0.994	0.10	very small
T2 fetal weight centiles	37.07 ± 31.00	34.85 ± 33.66	0.723	0.07	null
T3 fetal weight (g)	2,165.79 ± 347.20	2,048.92 ± 411.66	0.858	0.28	small
T3 fetal weight centiles	69.79 ± 22.00	59.88 ± 24.06	0.087	−0.10	small
T2 SCFT (mm)	3.27 ± 0.47	2.96 ± 0.52	**0.021**	**0.60**	**average**
T3 SCFT (mm)	4.41 ± 0.77	4.24 ± 0.88	0.053	0.19	very small
T2 total thigh tissue (cm^3^)	5.63 ± 1.34	5.28 ± 1.48	0.824	0.24	small
T3 total thigh tissue (cm^3^)	13.55 ± 2.56	13.40 ± 2.33	0.283	0.06	null
T2 thigh muscle mass (cm^3^)	3.29 ± 0.83	3.05 ± 0.87	1.000	0.28	small
T3 thigh muscle mass (cm^3^)	7.45 ± 1.47	7.51 ± 1.36	0.917	−0.04	null
T2 subcutaneous thigh fat (cm^3^)	2.37 ± 0.59	2.23 ± 0.67	0.382	0.21	small
T3 subcutaneous thigh fat (cm^3^)	6.10 ± 1.38	5.81 ± 1.23	0.118	0.24	small
T2 total arm tissue (cm3)	3.29 ± 0.93	2.82 ± 0.82	**0.033**	**0.57**	**average**
T3 total arm tissue (cm3)	7.31 ± 1.60	6.72 ± 1.52	0.713	0.39	small
T2 arm muscle mass (cm^3^)	1.73 ± 0.56	1.49 ± 0.50	**0.046**	0.48	small
T3 arm muscle mass (cm^3^)	3.61 ± 0.81	3.39 ± 0.70	0.561	0.31	small
T2 subcutaneous arm fat (cm^3^)	1.58 ± 0.42	1.33 ± 0.37	**0.029**	**0.68**	**average**
T3 subcutaneous arm fat (cm^3^)	3.71 ± 1.02	3.33 ± 1.00	0.565	0.38	small
**Newborns**					
T4 weight (g)	3,309.6 ± 439.30	3,189.32 ± 408.43	0.130	0.29	small
T4 length (cm)	48.36 ± 2.29	48.13 ± 2.30	0.137	0.10	very small
T4 head circumference (cm)	34.14 ± 1.44	33.42 ± 1.34	**0.046**	**0.54**	**average**
T4 thoracic circumference (cm)	33.51 ± 2.11	33.00 ± 1.90	0.097	0.27	small
T4 abdominal circumference (cm)	32.10 ± 2.25	31.02 ± 2.64	0.454	0.41	small
T4 BMI (kg/m^2^)	14.16 ± 1.90	13.73 ± 1.28	0.350	0.34	small

Mean ± SD. T-test for independent samples and Glass' delta to effect size. BMI, Body Mass Index; SCFT, abdominal subcutaneous fat thickness. T1 = ≤ 15 gestational weeks. T2 = 20–26 weeks. T3 = 30–36 weeks. T4 = delivery. Bold values indicate statistically significant difference.

**Figure 1:**
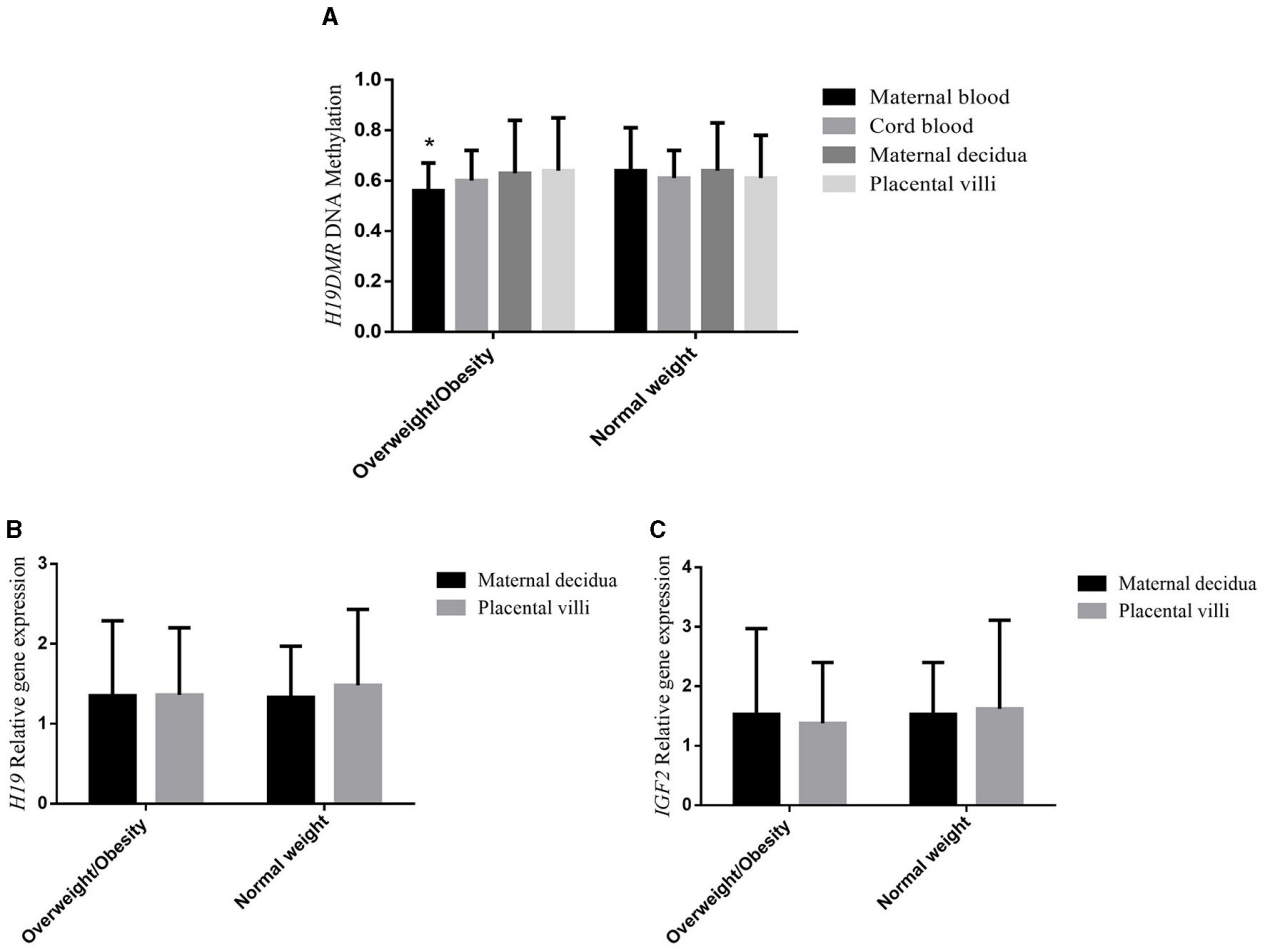
**(A)** *H19DMR* methylation, **(B)** *H19* gene expression, and **(C)** *IGF2* gene expression of overweight/obese, and normal weight pregnant women in maternal blood, cord blood, maternal decidua, and placental villi tissues. ANOVA with Bonferroni's post-hoc test. *p < 0.05.

The PCR-RFLP demonstrated that there were no variations in the homologous DNA sequences of the *H19DMR*, implying the absence of genetic bias. In samples that had variations, the final methylation percentages could be altered due to the gene polymorphism and not to the environmental factor of interest. Figure 1A shows the mean DNA methylation level of *H19DMR* in maternal blood, cord blood, maternal decidua and placental villi of the overweight/obese and normal weight groups. *H19DMR* was found to be significantly less methylated (*p* = 0.038) in maternal blood of the overweight/obese group compared to the normal weight group, but no significant difference between groups was observed for the other tissues (*p* = 0.553 cord blood; *p* = 0.344 placental villi; *p* = 0.608 maternal decidua). Figures 1B, C shows the mean relative gene expression of the *H19* and *IGF2* genes, respectively, in the maternal decidua and placental villi of the overweight/obese and normal weight groups. There was no difference in gene expression between groups (*p* = 0.568, *H19* placental villi; *p* = 0.705, *IGF2* placental villi; *p* = 0.705, *H19* maternal decidua; *p* = 0.234, *IGF2* maternal decidua). Bonferroni's *post-hoc* test also revealed no difference in methylation or gene expression in the different tissues between groups. There was a positive correlation between *H19DMR* methylation in placental villi and in maternal decidua (ρ = 0.788, *p* = 0.001), between *H19* and *IGF2* gene expression in maternal decidua (ρ = 0.841, *p* = 0.001), and between *H19* and *IGF2* gene expression in placental villi (ρ = 0.912, *p* = 0.001). Correlations of DNA methylation and gene expression with fetal and newborn parameters, and correlations of DNA methylation itself and with gene expression in different tissues, are shown in Supplementary Tables S2A, B, respectively.

Table 3 shows the associations of mean *H19DMR* methylation level in different tissues with fetal growth and body composition and newborn anthropometric parameters. There were significant associations of BPD centiles at T2 with methylation in cord blood (*p* = 0.026); OFD at T3 with methylation in placental villi (*p* = 0.001) and in maternal decidua (*p* = 0.019); OFD centiles at T3 with methylation in placental villi (*p* = 0.001) and in maternal decidua (*p* = 0.023); HC at T2 with methylation in placental villi (*p* = 0.004) and in maternal decidua (*p* = 0.041); HC at T3 with methylation in placental villi (*p* = 0.009); HC centiles at T3 with methylation in placental villi (*p* = 0.007); length at T2 with methylation in maternal decidua (*p* = 0.050); SCFT at T3 with methylation in cord blood (*p* = 0.004) and in placental villi (*p* = 0.037), and HC at T4 with methylation in cord blood (*p* = 0.016) and in placental villi (*p* = 0.020).

**Table 3:** Multiple linear regression models showing the associations of mean *H19DMR* methylation in maternal blood, cord blood, maternal decidua and placental villi tissues with fetal and newborn parameters.

**DNA methylation**	**β**	***r* ^2^**	***p***	**95% CI**
**T2 Biparietal diameter centiles**				
***H19DMR*** **cord blood methylation**	87.208	0.153	**0.026**	10.917; 163.498
Pre-pregnancy BMI	1.139		0.331	−1.192; 3.470
Gestational weight gain	−0.538		0.538	−2.282; 1.206
Maternal age	0.220		0.754	−1.179; 1.619
Newborn sex	8.066		0.386	−10.440; 26.572
Gestational age in T2	−0.640		0.785	−5.323; 4.044
**T3 occipitofrontal diameter**				
***H19DMR*** **placental villi methylation**	1.339	0.325	**0.001**	0.594; 2.084
Pre-pregnancy BMI	0.002		0.920	−0.036; 0.039
Gestational weight gain	−0.009		0.510	−0.038; 0.019
Maternal age	0.007		0.554	−0.016; 0.030
Newborn sex	−0.038		0.796	−0.335; 0.258
Gestational age in T3	0.172		0.001	0.074; 0.270
***H19DMR*** **maternal decidua methylation**	0.902	0.248	**0.019**	0.151;1.654
Pre-pregnancy BMI	0.003		0.898	−0.037; 0.042
Gestational weight gain	−0.003		0.839	−0.033; 0.027
Maternal age	0.005		0.660	−0.019; 0.029
Newborn sex	−0.025		0.874	−0.338; 0.288
Gestational age in T3	0.174		0.001	0.070; 0.277
**T3 occipitofrontal diameter centiles**				
***H19DMR*** **placental villi methylation**	74.088	0.209	**0.001**	32.055; 116.120
Pre-pregnancy BMI	0.415		0.696	−1.701; 2.530
Gestational weight gain	−0.045		0.956	−1.657; 1.566
Maternal age	0.929		0.153	−0.356; 2.215
Newborn sex	−3.470		0.680	−20.206; 13.266
Gestational age in T3	−2.274		0.414	−7.811; 3.263
***H19DMR*** **maternal decidua methylation**	49.334	0.120	**0.023**	6.976; 91.693
Pre-pregnancy BMI	0.454		0.685	−1.779; 2.688
Gestational weight gain	0.316		0.707	−1.358; 1.990
Maternal age	0.846		0.216	−0.508; 2.201
Newborn sex	−2.717		0.759	−20.366; 14.931
Gestational age in T3	−2.185		0.457	−8.026; 3.656
**T2 head circumference**				
***H19DMR*** **placental villi methylation**	4.416	0.517	**0.004**	1.437; 7.395
Pre-pregnancy BMI	−0.014		0.858	−0.165; 0.137
Gestational weight gain	−0.132		0.025	−0.247; −0.017
Maternal age	−0.042		0.353	−0.133; 0.048
Newborn sex	−0.069		0.909	−1.271; 1.133
Gestational age in T2	0.972		0.000	0.657; 1.286
***H19DMR*** **maternal decidua methylation**	3.088	0.482	**0.041**	0.127; 6.049
Pre-pregnancy BMI	−0.013		0.866	−0.170; 0.144
Gestational weight gain	−0.109		0.068	−0.225; 0.008
Maternal age	−0.046		0.328	−0.140; 0.048
Newborn sex	0.015		0.980	−1.230; 1.261
Gestational age in T2	1.037		0.000	0.710; 1.364
**T3 head circumference**				
***H19DMR*** **placental villi methylation**	1.989	0.456	**0.009**	0.517; 3.461
Pre-pregnancy BMI	0.020		0.600	−0.055; 0.094
Gestational weight gain	0.004		0.881	−0.052; 0.061
Maternal age	0.020		0.382	−0.025; 0.065
Newborn sex	0.102		0.729	−0.484; 0.688
Gestational age in T3	0.578		0.000	0.384; 0.772
**T3 head circumference centiles**				
***H19DMR*** **placental villi methylation**	55.988	0.161	**0.007**	15.677; 96.299
Pre-pregnancy BMI	0.574		0.574	−1.455; 2.603
Gestational weight gain	0.270		0.727	−1.275; 1.816
Maternal age	0.669		0.282	−0.564; 1.902
Newborn sex	0.450		0.955	−15.601; 16.500
Gestational age in T3	−2.578		0.335	−7.889; 2.732
**T2 length**				
***H19DMR*** **maternal decidua methylation**	2.002	0.829	**0.050**	−0.007; 4.011
Pre-pregnancy BMI	−0.042		0.437	−0.148; 0.065
Gestational weight gain	−0.085		0.036	−0.164; −0.006
Maternal age	0.023		0.475	−0.041; 0.086
Newborn sex	0.003		0.995	−0.842; 0.847
Gestational age in T2	1.720		0.000	1.498; 1.941
**T3 abdominal subcutaneous fat thickness**				
***H19DMR*** **cord blood methylation**	2.445	0.331	**0.004**	0.812; 4.077
Pre-pregnancy BMI	−0.011		0.651	−0.061; 0.039
Gestational weight gain	−0.040		0.038	−0.077; −0.002
Maternal age	−0.001		0.944	−0.032; 0.030
Newborn sex	0.509		0.012	0.116; 0.903
Gestational age in T3	0.176		0.008	0.047; 0.305
***H19DMR*** **placental villi methylation**	−1.093	0.218	**0.037**	−2.117; −0.068
Pre-pregnancy BMI	0.006		0.802	−0.045; 0.058
Gestational weight gain	−0.000		0.997	−0.039; 0.039
Maternal age	0.015		0.346	−0.016; 0.046
Newborn sex	0.330		0.111	−0.078; 0.738
Gestational age in T3	0.158		0.023	0.023; 0.293
***H19DMR*** **cord blood methylation**	3.340	0.333	**0.016**	0.666; 6.015
Pre-pregnancy BMI	−0.010		0.823	−0.096; 0.076
Gestational weight gain	−0.021		0.505	−0.082; 0.041
Maternal age	0.028		0.265	−0.022; 0.078
Newborn sex	0.334		0.297	−0.304; 0.972
Gestational age in T4	0.356		0.000	0.175; 0.538
***H19DMR*** **placental villi methylation**	1.827	0.316	**0.023**	0.258; 3.397
Pre-pregnancy BMI	−0.017		0.679	−0.101; 0.066
Gestational weight gain	−0.025		0.395	−0.085; 0.034
Maternal age	0.025		0.304	−0.023; 0.072
Newborn gender	0.351		0.254	−0.260; 0.962
Gestational age in T4	0.302		0.002	0.120; 0.485

BMI, Body Mass Index. T1 = ≤ 15 gestational weeks. T2 = 20–26 weeks. T3 = 30–36 weeks. T4 = delivery. Bold values indicate statistically significant difference.

We also found significant associations (Table 4) of the mean *H19* and *IGF2* gene expression levels in different tissues with fetal growth and body composition and newborn anthropometric parameters: BPD at T3 with *H19* expression in maternal decidua (*p* = 0.018) and with *IGF2* expression in maternal decidua (*p* = 0.011); BPD centiles at T3 with *H19* expression in maternal decidua (*p* = 0.011) and with *IGF2* expression in maternal decidua (*p* = 0.007); OFD at T2 with *H19* (*p* = 0.022) and *IGF2* expression in placental villi (*p* = 0.016); OFD centiles at T2 with *H19* (*p* = 0.006) and *IGF2* expression in placental villi (*p* = 0.005); FL centiles at T2 with *H19* expression in maternal decidua (*p* = 0.041), and subcutaneous arm fat at T3 with *H19* expression in placental villi (*p* = 0.033).

**Table 4:** Multiple linear regression models showing the associations of mean *H19* and *IGF2* gene expression in maternal decidua and placental villi tissues with fetal and newborn parameters.

**Gene expression**	**β**	**r^2^**	***p***	**95% CI**
**T3 biparietal diameter**				
***H19*** **maternal decidua expression**	0.120	0.567	**0.018**	0.021; 0.219
Pre-pregnancy BMI	0.008		0.442	−0.012; 0.027
Gestational weight gain	0.013		0.074	−0.001; 0.027
Maternal age	0.006		0.310	−0.006; 0.018
Newborn sex	0.177		0.026	0.022; 0.333
Gestational age in T3	0.210		0.000	0.157; 0.262
***IGF2*** **maternal decidua expression**	0.088	0.574	**0.011**	0.021; 0.155
Pre-pregnancy BMI	0.007		0.476	−0.012; 0.026
Gestational weight gain	0.013		0.066	−0.001; 0.027
Maternal age	0.005		0.349	−0.006; 0.017
Newborn sex	0.170		0.030	0.017; 0.323
Gestational age in T3	0.212		0.000	0.160; 0.265
**T3 biparietal diameter centiles**				
***H19*** **maternal decidua expression**	11.412	0.219	**0.011**	2.755; 20.069
Pre-pregnancy BMI	0.551		0.519	−1.151; 2.254
Gestational weight gain	0.889		0.156	−0.350; 2.128
Maternal age	0.385		0.455	−0.641; 1.410
Newborn sex	15.772		0.024	2.157; 29.387
Gestational age in T3	1.150		0.619	−3.459; 5.759
***IGF2*** **maternal decidua expression**	8.191	0.229	**0.007**	2.325; 14.057
Pre-pregnancy BMI	0.501		0.556	−1.194; 2.196
Gestational weight gain	0.916		0.142	−0.315; 2.148
Maternal age	0.338		0.510	−0.682; 1.358
Newborn sex	15.025		0.029	1.602; 28.447
Gestational age in T3	1.360		0.556	−3.243; 5.964
**T2 occipitofrontal diameter**				
***H19*** **placental villi expression**	−0.102	0.820	**0.022**	−0.189; −0.015
Pre-pregnancy BMI	0.008		0.470	−0.014; 0.029
Gestational weight gain	−0.014		0.077	−0.029; 0.002
Maternal age	−0.014		0.030	−0.027; −0.001
Newborn sex	0.122		0.155	−0.048; 0.291
Gestational age in T2	0.326		0.000	0.281; 0.370
***IGF2*** **placental villi expression**	−0.073	0.822	**0.016**	−0.132; −0.014
Pre-pregnancy BMI	0.008		0.449	−0.013; 0.029
Gestational weight gain	−0.014		0.067	−0.030; 0.001
Maternal age	−0.015		0.018	−0.028; −0.003
Newborn sex	0.114		0.179	-.054; 0.283
Gestational age in T2	0.327		0.000	0.283; 0.371
**T2 occipitofrontal diameter centiles**				
***H19*** **placental villi expression**	−10.734	0.324	**0.006**	−18.193; −3.276
Pre-pregnancy BMI	0.569		0.540	−1.280; 2.419
Gestational weight gain	−1.053		0.122	−2.395; 0.290
Maternal age	−1.055		0.059	−2.154; 0.043
Newborn sex	10.567		0.157	−4.207; 25.341
Gestational age in T2	−6.174		0.002	−9.995; −2.352
***IGF2*** **placental villi expression**	−7.457	0.327	**0.005**	−12.536; −2.378
Pre-pregnancy BMI	0.593		0.522	−1.252; 2.437
Gestational weight gain	−1.105		0.104	−2.445; 0.235
Maternal age	−1.196		0.034	−2.297; −0.095
Newborn sex	9.901		0.183	−4.827; 24.630
Gestational age in T2	−6.018		0.002	−9.822; −2.215
**T2 femur length centiles**				
***H19*** **maternal decidua expression**	−9.010	0.138	**0.041**	−17.659; −0.361
Pre-pregnancy BMI	−0.356		0.689	−2.131; 1.418
Gestational weight gain	−1.368		0.037	−2.652; −0.084
Maternal age	−0.133		0.800	−1.182; 0.916
Newborn sex	−4.121		0.565	−18.398; 10.156
Gestational age in T2	−1.017		0.583	−4.710; 2.676
**T3 subcutaneous arm fat**				
***H19*** **placental villi expression**	0.262	0.331	**0.033**	0.022; 0.503
Pre-pregnancy BMI	0.027		0.360	−0.031; 0.084
Gestational weight gain	0.007		0.741	−0.035; 0.049
Maternal age	0.002		0.929	−0.033; 0.036
Newborn sex	−0.192		0.403	−0.648; 0.264
Gestational age in T3	0.423		0.000	0.269; 0.577

BMI, Body Mass Index. T1 = ≤ 15 gestational weeks. T2 = 20–26 weeks. T3 = 30–36 weeks. T4 = delivery. Bold values indicate statistically significant difference.

## 4. Discussion

In this study we seek to understand how maternal overweight/obesity affects DNA methylation in an important ICR and expression of genes possibly regulated by this ICR in different tissues. Furthermore, we wanted to understand how methylation of *H19DMR* and expression of the *H19* and *IGF2* genes are associated with the fetal and newborn parameters assessed in this study.

Studies have investigated the role of maternal overweight/obesity in fetal metabolic programming. BMI was found to be associated with changes in DNA methylation in genetic loci in adults, as well as with the level of methylation at CpGs sites (21). However, to our knowledge, there are no prospective studies on the relationship between maternal and fetal genetic information of *H19DMR, H19* and *IGF2* in different maternal-fetal tissues and fetal body composition. It is known that the higher the BMI, the greater the risk of developing metabolic diseases, especially gestational diabetes and hypertension (22). Despite these risks, we did not find significant numbers of pregnant women who became diabetic or hypertensive, or who had any chronic diseases during pregnancy. In the present study, pregnant women in the overweight/obese group had a higher pre-pregnancy BMI and maintained a high BMI at all time points until the end of pregnancy when compared to pregnant women with a normal pre-pregnancy BMI.

There was no difference in age, race, education, parity or lifestyle between overweight/obese and normal weight pregnant women, considering that both groups were healthy and young. However, one parameter that differed between groups was the sex of the newborn, with a larger number of male newborns in the overweight/obese group and of female newborns in the normal weight group. The literature suggests a relationship between sex and body composition at birth (23) and we therefore chose to evaluate the variables also as centiles since this approach takes into account sex. In addition, we included newborn sex as a confounding variable in the multiple linear regression models. We found higher HC centiles at T2 and higher BPD and OFD centiles at T3 in the overweight/obese group.

The NICHD Fetal Growth Studies-Singletons assessed the association between maternal obesity and longitudinal measurements of fetal growth. Similar to our results the authors observed higher HC in fetuses born to obese women compared to fetuses in the non-obese group, but no difference for fetal BPD. However, this difference occurred between the 33rd and 35th gestational week, while in our study this difference was observed at an earlier time (T2) and at birth (T4) (24).

In addition to biometric parameters for investigating fetal growth, we also used ultrasound to assess fetal body composition. The development of adipose tissue starts to become more important by the 24th week and increases rapidly in the third trimester of gestation (25). Body mass is defined by ultrasound as a two-compartment model that considers fat mass and lean mass as areas of interest. Body tissues are quantified by analyzing the thickness of the subcutaneous tissue; however, a limitation of 2D ultrasound is the inability to characterize visceral fat deposits, which can be of metabolic importance during the fetal period and other periods of life (26).

Regarding fetal body composition, our findings were more specific at T2. At this time point, adipose parameters such as SCFT and fetal subcutaneous arm fat, as well as body composition parameters such as total arm tissue and arm muscle mass, were higher in the group of overweight/obese women. Studies have shown that SCFT can predict fetal macrosomia, SCFT values higher than 6.25 mm were sensitive in predicting large-for-gestational-age babies (27). In our study, SCFT did not remain elevated at T3 and the highest value found was 4.41 mm in the group of overweight/obese pregnant women at T3; thus, our babies were not born with macrosomia. Although ultrasound measurements few days before delivery are important, we do not have such measurements. However, the newborns of this study were not large-for-gestational age, although newborns in the overweight/obese group were on average 120 g heavier than those in the normal weight group.

A retrospective study that investigated SCFT from the second trimester to delivery showed higher values in obese pregnant women (pre-pregnancy BMI 35.4 ± 5.1 kg/m^2^) compared to non-obese women (pre-pregnancy BMI 29.6 ± 4.4 kg/m^2^) (28). In our study, overweight and obese women were selected according to their pre-pregnancy BMI and the difference in fat mass between overweight/obese and normal weight pregnant women was significant (15.64 vs. 10.67 kg; *p* = 0.007). An important fact is that gestational weight gain was similar in the two groups. Therefore, we mainly evaluated the impact of pre-pregnancy BMI and not of weight gain on the fetal and newborn parameters. Within this context, high maternal gestational weight gain can increase nutrient availability to the fetus, with consequent changes in fetal growth parameters and adiposity. However, in this study, although not always significant, all parameters were higher in offspring of overweight/obese pregnant women.

Regarding the methylation of *H19DMR* in different maternal (maternal peripheral blood and maternal decidua) and fetal (cord blood and placental villi) tissues, we found that this ICR was less methylated in maternal blood of obese/overweight pregnant women compared to the normal weight group. There was no difference in methylation between groups in the other tissues. The hypomethylation of *H19* was described in intrauterine growth restriction which, in turn, would affect the biological potential of fetal growth (29). The lower methylation in maternal blood did not negatively regulate fetal or newborn growth in this study. Tabano et al. reported a minimum percentage of *H19DMR* methylation of 44% in peripheral blood of healthy human adults (30). In our study, lower methylation was observed in the peripheral blood of overweight/obese women (mean level 56%) compared to the normal weight group. However, we emphasize that such values are not directly comparable, since the method used for methylation detection was semiquantitative, while other studies use quantitative methods, such as pyrosequencing. Thus, we therefore believe that this result did not cause a negative impact on newborn growth in this group.

The *H19* gene has been suggested to play a role in cancer pathogenesis and is involved in genetic syndromes, as well as in maternal epigenetics and fetal and neonatal growth. Its role in the maternal metabolic and dietary context has also been studied (31). Thus, in addition to weight, maternal metabolic and dietary data would contribute to a better understanding of the modifications of *H19DMR* in maternal blood. The Newborn Epigenetics Study (NEST) cohort demonstrated higher estimated mean *H19DMR* methylation levels in cord blood of newborns from obese mothers compared to non-obese mothers (32). Newborns with weight-for-age > 85th centiles had higher *H19DMR* methylation levels in cord blood than newborns with weight-for-age <85th centiles (7).

In genomic imprinting, only one allele is expressed in the case of certain genes. In humans, a group of genes regulated by genomic imprinting was mapped to the short arm of chromosome 11, in the 11p15.5 region. Two imprinting control regions, one of which is ICR1 that, among other genes, harbors the *IGF2* and *H19* tumor suppressor genes, were also mapped. ICR1 is methylated on the paternal allele and is regulated by the *H19* gene. In the absence of methylation on the maternal allele, enhancers can access the 3′-end of the *H19* gene, enabling the binding of a protein or CCCTC-binding factor (CTCF) to ICR1. The CTCF protein acts as an insulator, blocking the access of enhancers to the 3′-end of the *IGF2* gene. In the maternal allele, the *H19* gene is expressed and the *IGF2* gene is silenced. Methylation on the paternal allele blocks the binding of the CTCF protein to ICR1, inhibiting its insulator function and allowing access of enhancers to the 3′-end of the *IGF2* gene, with consequent expression of this gene and silencing of *H19*. The expression of imprinted genes is facilitated by asymmetric epigenetic markers on the maternal or paternal allele, forming clusters that contain DMRs. One of the most studied markers is DNA methylation (10). In addition, *H19* is a non-coding RNA, ultimately it would block the transcription of *IGF2* tending to reduce fetal growth, but not producing a protein. We emphasize that in this study the region that we call *H19DMR* is located within ICR1.

We therefore also analyzed the gene expression of *H19* and *IGF2* in placental villi and maternal decidua; however, we did not find a difference in the expression of these genes between overweight/obese and normal weight women. The same was observed for tissue analysis, with no difference in *H19DMR* methylation in cord blood, or placental villi and maternal decidua. However, correlation analysis showed strong positive correlations between *H19DMR* methylation in placental villi and in maternal decidua, between *H19* and *IGF2* gene expression in maternal decidua, and between *H19* and *IGF2* gene expression in placental villi. In other words, this study shows a positive correlation between the two tissues studied (maternal decidua and placental villi), with similar patterns of methylation and gene expression.

Despite the theoretical understanding of the genomic imprinting of *H19* and *IGF2*, the presence of contrasting results in the literature indicates that there is not always a direct correlation between methylation and the expression of imprinted genes; thus, other mechanisms may be involved in this pathway. Methylation of the *IGF2* and *H19* promoters is not a prerequisite for the regulation of the imprinting domain that controls *IGF2* and *H19* transcription in the human placenta (33). Extraembryonic hypomethylation of *H19* had no impact on the expression pattern of *IGF2* or *H19* (30).

In our study, the methylation of *H19DMR* in cord blood was associated with the centiles of BPD at T2, SCFT at T3, and HC at T4. The methylation of *H19DMR* in maternal decidua was associated with OFD at T3, OFD centiles at T3, HC at T2, and length at T2. The methylation of *H19DMR* in placental villi was associated with OFD at T3, OFD centiles at T3, HC at T2 and T3, HC centiles at T3, SCFT at T3, and HC at T4. We also found an association of *H19* gene expression in maternal decidua with BPD at T3, BPD centiles at T3, and FL centiles at T2, as well as an association of *H19* expression in placental villi with OFD at T2, OFD centiles at T2, and subcutaneous arm fat at T3. *IGF2* gene expression in maternal decidua was associated with BPD at T3 and BPD centiles. In addition, there was an association of *IGF2* gene expression in placental villi with OFD at T2 and OFD centiles at T2. A study using term placenta and chorionic villus samples found that *IGF2* expression in term placentas was not correlated with fetal growth parameters, while *IGF2* expression in chorionic villus samples was correlated with crown rump length and birth weight (5). Another study analyzed the effect of maternal factors on the methylation of *H19DMR* in cord blood and also found changes in DNA methylation to be associated with HC (34).

In the present study, we highlight that: high pre-pregnancy BMI regulated *H19DMR* methylation in maternal blood and altered some parameters of fetal and newborn growth and fetal adiposity; *IGF2* and *H19* gene expression did not seem to be the link between obesity and the offspring outcomes investigated; and *H19DMR* methylation and expression of *IGF2* and *H19* genes in cord blood and especially in maternal decidua and placental villi regulated several parameters of fetal growth and body composition. To our knowledge, this is the first study to demonstrate associations of imprinted genes variations at the maternal-fetal interface of the placenta and in cord blood with fetal body composition, supporting the involvement of epigenetic mechanisms in offspring growth and body composition.

Strengths of our study include specific evaluation of fetal adiposity by ultrasonography and assessment of maternal blood and cord blood methylation, and maternal decidua and placental villi tissues methylation and gene expression. Limitations of this study were the non-determination of gene expression in maternal and cord blood, the small sample size, and the semi-quantitative method for assessing DNA methylation.

## Data availability statement

The raw data supporting the conclusions of this article will be made available by the authors, upon request.

## Ethics statement

The studies involving humans were approved by Research Ethics Committee of the School of Public Health, University of São Paulo (protocol number 2.570.576). This study was conducted in compliance with the Helsinki guidelines. The studies were conducted in accordance with the local legislation and institutional requirements. Written informed consent for participation in this study was provided by the participants' legal guardians/next of kin. Written informed consent was obtained from the individual (s), and minor (s)' legal guardian/next of kin, for the publication of any potentially identifiable images or data included in this article.

## Author contributions

PR, PA, ER, and JM designed the research. PR coordinated the field work. PA, PN, LT, LB, ML, and LL participated in the collection of clinical data. PA and ND participated in the collection of biological material. PA, JM, and ER performed the experiments, analysis, and interpretation of DNA methylation and expression genic data. PA and PR directed statistical analysis. PA and JM wrote the first draft of the manuscript. PR and ER reviewed the manuscript and provided critical revision. All authors read and approved the final manuscript.

## Supplementary material

Supplementary Figure S1 Amplification region of the *H19DMR* primers.

Supplementary Table S1 Methylation primers and gene expression.

Supplementary Table S2 **(A)** Correlations of mean *H19DMR* methylation in maternal blood, cord blood, maternal decidua and placental villi tissues with fetal and newborn parameters. **(B)** Correlation of DNA methylation itself with gene expression in maternal blood, cord blood, maternal decidua and placental villi tissues.
