# Supplementary figures and images for: The relationship between obesity-related H19DMR methylation and H19 and IGF2 gene expression on offspring growth and body composition
Source: Front Nutr. 2023 Sep 21;10:1170411. doi: 10.3389/fnut.2023.1170411 (PMC10552537; doi:10.3389/fnut.2023.1170411)

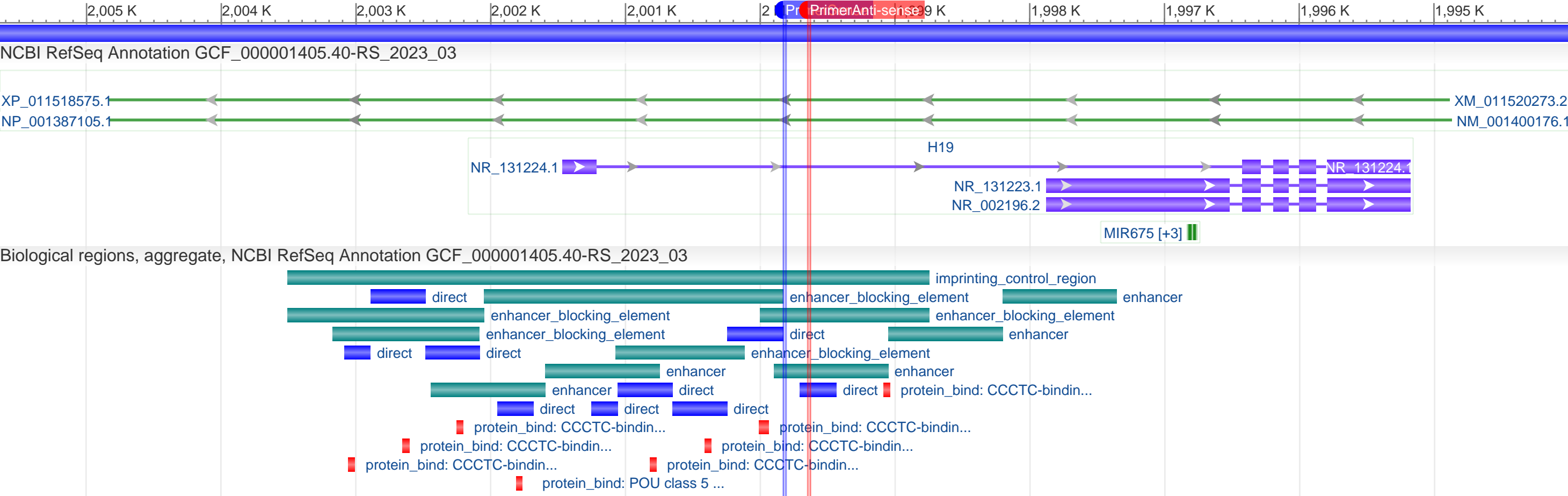

Supplement: Supplementary Figure S1 — Amplification region of the H19DMR primers. [file Data_Sheet_1.PDF]
