# Supplementary material for: The relationship between obesity-related H19DMR methylation and H19 and IGF2 gene expression on offspring growth and body composition
Source: Front Nutr. 2023 Sep 21;10:1170411. doi: 10.3389/fnut.2023.1170411 (PMC10552537; doi:10.3389/fnut.2023.1170411)
Supplement: Supplementary Table S1 — Methylation primers and gene expression. [file Table_1.DOCX]

**Table S1**. Sequences of primers

| **Gene** | **Guidance** | **Sequence 5’- 3’** | **Bank access number** |
| --- | --- | --- | --- |
| **Methylation** | | | |
| *H19DMR* | *Sense* | ATCTTCAGGTCGGGCATTATCC | AF125183.1 |
|  | *Anti-sense* | CAGTTCAGTAAAAGGCTGGGGA | GenBank NCBI |
| **Gene expression** | | | |
| *YWHAZ* | *Sense* | TTGGAGGGTCGTCTCAAGTATT | ENST00000395957.6 |
| *YWHAZ* | *Anti-sense* | TCTGATAGGATGTGTTGGTTGC | ENSEMBL |
| *GAPDH* | *Sense* | GATGACATCAAGAAGGTGGTGA | ENST00000229239.10 |
| *GAPDH* | *Anti-sense* | CAAATTCGTTGTCATACCAGGA | ENSEMBL |
| *IGF2* | *Sense* | GTGCTACCCCCGCCAAGT | ENST00000416167.7 |
| *IGF2* | *Anti-sense* | TGGACTGCTTCCAGGTGTCA | ENSEMBL |
| *H19* | *Sense* | GGCTCCCAGAACCCACAAC | ENST00000414790.6 |
| *H19* | *Anti-sense* | AGAGGGTTTTGTGTCCGGATT | ENSEMBL |
