# Supplementary material for: The relationship between obesity-related H19DMR methylation and H19 and IGF2 gene expression on offspring growth and body composition
Source: Front Nutr. 2023 Sep 21;10:1170411. doi: 10.3389/fnut.2023.1170411 (PMC10552537; doi:10.3389/fnut.2023.1170411)
Supplement: Supplementary Table S2 — (A) Correlations of mean H19DMR methylation in maternal blood, cord blood, maternal decidua and placental villi tissues with fetal and newborn parameters. (B) Correlation of DNA methylation itself with gene expression in maternal blood, cord blood, maternal decidua and placental villi tissues. [file Table_2.docx]

| **Table S2(A)**. Correlations of mean *H19DMR* methylation in maternal blood, cord blood, maternal decidua and placental villi tissues with fetal and newborn parameters. | | | | | | | | |
| --- | --- | --- | --- | --- | --- | --- | --- | --- |
|  | Maternal Blood *H19DMR*  Methylation | Cord  Blood  *H19DMR*  Methylation | Placental  Villi  *H19DMR* Methylation | Maternal Decidua *H19DMR* Methylation | Placental Villi  *H19* Expression | Placental Villi  *IGF2*  Expression | Maternal Decidua  *H19* Expression | Maternal Decidua  *IGF2* Expression |
| T2 Fetal Weight | 0.073 | -0.135 | 0.022 | -0.094 | -0.152 | -0.191 | -0.177 | -0.314 |
|  | 0.595 | 0.307 | 0.866 | 0.464 | 0.235 | 0.134 | 0.166 | 0.012 |
| T2 Fetal Weight Centiles | 0.100 | -0.193 | -0.027 | -0.094 | -0.014 | -0.067 | -0.220 | -0.205 |
|  | 0.472 | 0.147 | 0.836 | 0.469 | 0.915 | 0.607 | 0.086 | 0.110 |
| T3 Fetal Weight | 0.160 | 0.020 | 0.178 | 0.103 | -0.190 | -0.166 | -0.324 | -0.324 |
|  | 0.238 | 0.878 | 0.160 | 0.418 | 0.133 | 0.190 | 0.009 | 0.009 |
| T3 Fetal Weight Centiles | 0.143 | 0.207 | 0.184 | 0.032 | -0.071 | -0.052 | -0.185 | -0.083 |
|  | 0.292 | 0.113 | 0.145 | 0.802 | 0.576 | 0.686 | 0.143 | 0.513 |
| T4 Weight | 0.070 | 0.118 | 0.149 | 0.014 | -0.022 | 0.044 | 0.095 | 0.142 |
|  | 0.607 | 0.367 | 0.240 | 0.911 | 0.862 | 0.731 | 0.457 | 0.265 |
| T2 Biparietal Diameter | 0.096 | -0.028 | 0.005 | -0.117 | -0.048 | -0.121 | -0.082 | -0.252 |
|  | 0.484 | 0.834 | 0.969 | 0.363 | 0.707 | 0.345 | 0.521 | 0.046 |
| T2 Biparietal Diameter Centiles | 0.068 | 0.227 | 0.048 | 0.003 | 0.217 | 0.140 | 0.115 | 0.125 |
|  | 0.626 | 0.087 | 0.710 | 0.980 | 0.091 | 0.276 | 0.373 | 0.334 |
| T3 Biparietal Diameter | 0.174 | 0.083 | 0.006 | -0.011 | 0.017 | -0.002 | -0.091 | -0.067 |
|  | 0.200 | 0.526 | 0.963 | 0.930 | 0.896 | 0.990 | 0.477 | 0.600 |
| T3 Biparietal Diameter Centiles | 0.096 | 0.166 | -0.050 | -0.064 | 0.247 | 0.177 | 0.143 | 0.181 |
|  | 0.480 | 0.205 | 0.697 | 0.616 | 0.049 | 0.162 | 0.260 | 0.151 |
| T2 Occipitofrontal Diameter | -0.039 | -0.113 | 0.017 | -0.107 | -0.285 | -0.312 | -0.196 | -0.322 |
|  | 0.776 | 0.394 | 0.896 | 0.403 | 0.023 | 0.013 | 0.123 | 0.010 |
| T2 Occipitofrontal Diameter Centiles | -0.221 | 0.060 | 0.061 | 0.132 | -0.223 | -0.207 | -0.080 | 0.030 |
|  | 0.108 | 0.655 | 0.636 | 0.307 | 0.081 | 0.107 | 0.535 | 0.814 |
| T3 Occipitofrontal Diameter | 0.041 | -0.013 | 0.383 | 0.270 | 0.039 | 0.006 | -0.147 | -0.168 |
|  | 0.763 | 0.922 | 0.002 | 0.031 | 0.759 | 0.961 | 0.245 | 0.184 |
| T3 Occipitofrontal Diameter Centiles | -0.022 | 0.044 | 0.406 | 0.293 | 0.160 | 0.103 | -0.048 | -0.037 |
|  | 0.874 | 0.740 | 0.001 | 0.019 | 0.205 | 0.417 | 0.709 | 0.769 |
| T2 Head Circumference | -0.006 | -0.081 | 0.086 | -0.055 | -0.155 | -0.173 | -0.116 | -0.240 |
|  | 0.965 | 0.543 | 0.504 | 0.670 | 0.224 | 0.176 | 0.364 | 0.058 |
| T2 Head Circumference Centiles | -0.118 | 0.169 | 0.129 | 0.151 | -0.018 | 0.019 | 0.012 | 0.118 |
|  | 0.398 | 0.209 | 0.324 | 0.246 | 0.889 | 0.886 | 0.924 | 0.366 |
| T3 Head Circumference | 0.090 | 0.006 | 0.272 | 0.189 | 0.036 | 0.004 | -0.167 | -0.166 |
|  | 0.510 | 0.965 | 0.029 | 0.134 | 0.775 | 0.976 | 0.186 | 0.190 |
| T3 Head Circumference Centiles | -0.020 | 0.064 | 0.350 | 0.245 | 0.238 | 0.180 | 0.012 | 0.058 |
|  | 0.884 | 0.629 | 0.005 | 0.051 | 0.059 | 0.154 | 0.926 | 0.648 |
| T4 Head Circumference | 0.007 | 0.048 | 0.152 | 0.013 | -0.176 | -0.149 | -0.050 | 0.058 |
|  | 0.961 | 0.715 | 0.232 | 0.920 | 0.164 | 0.239 | 0.696 | 0.649 |
| T2 Abdominal Circumference | -0.054 | -0.165 | -0.040 | -0.175 | -0.087 | -0.148 | -0.143 | -0.233 |
|  | 0.693 | 0.213 | 0.757 | 0.171 | 0.496 | 0.247 | 0.264 | 0.066 |
| T2 Fetal Abdominal Circumference Centiles | 0.022 | -0.095 | -0.023 | -0.014 | 0.072 | 0.041 | -0.171 | 0.050 |
|  | 0.878 | 0.482 | 0.861 | 0.917 | 0.582 | 0.752 | 0.188 | 0.700 |
| T3 Abdominal Circumference | 0.199 | 0.076 | 0.133 | 0.045 | -0.252 | -0.196 | -0.283 | -0.287 |
|  | 0.142 | 0.564 | 0.294 | 0.725 | 0.045 | 0.120 | 0.023 | 0.022 |
| T3 Fetal Abdominal Circumference Centiles | 0.131 | 0.162 | 0.073 | -0.045 | -0.147 | -0.089 | -0.111 | -0.078 |
|  | 0.337 | 0.217 | 0.565 | 0.726 | 0.247 | 0.485 | 0.383 | 0.538 |
| T4 Abdominal Circumference | 0.128 | 0.113 | 0.207 | 0.051 | 0.086 | 0.135 | 0.080 | 0.225 |
|  | 0.348 | 0.389 | 0.100 | 0.690 | 0.499 | 0.286 | 0.532 | 0.074 |
| T2 Femur Length | 0.046 | -0.152 | 0.054 | -0.060 | -0.235 | -0.246 | -0.178 | -0.309 |
|  | 0.740 | 0.249 | 0.674 | 0.638 | 0.063 | 0.052 | 0.162 | 0.014 |
| T2 Femur Length Centiles | -0.069 | -0.251 | -0.012 | 0.089 | -0.236 | -0.227 | -0.189 | -0.091 |
|  | 0.619 | 0.057 | 0.927 | 0.491 | 0.065 | 0.076 | 0.142 | 0.481 |
| T3 Femur Length | 0.093 | -0.023 | 0.127 | 0.090 | -0.142 | -0.124 | -0.292 | -0.346 |
|  | 0.494 | 0.860 | 0.318 | 0.480 | 0.265 | 0.327 | 0.019 | 0.005 |
| T3 Femur Length Centiles | 0.061 | 0.004 | 0.208 | 0.160 | -0.105 | -0.103 | -0.231 | -0.261 |
|  | 0.657 | 0.974 | 0.099 | 0.207 | 0.408 | 0.418 | 0.066 | 0.037 |
| T2 Humeral Length | 0.051 | -0.111 | 0.031 | -0.137 | -0.219 | -0.202 | -0.159 | -0.306 |
|  | 0.709 | 0.402 | 0.808 | 0.284 | 0.085 | 0.113 | 0.214 | 0.015 |
| T3 Humeral Length | 0.102 | -0.102 | 0.107 | 0.007 | -0.091 | -0.035 | -0.237 | -0.229 |
|  | 0.455 | 0.440 | 0.400 | 0.956 | 0.474 | 0.784 | 0.059 | 0.068 |
| T2 SCFT | 0.149 | -0.048 | 0.132 | 0.056 | -0.057 | -0.124 | -0.003 | -0.068 |
|  | 0.283 | 0.721 | 0.306 | 0.664 | 0.660 | 0.337 | 0.980 | 0.599 |
| T3 SCFT | 0.261 | 0.314 | -0.261 | -0.257 | -0.037 | 0.009 | 0.004 | 0.027 |
|  | 0.052 | 0.015 | 0.037 | 0.040 | 0.774 | 0.945 | 0.974 | 0.830 |
| T2 Total Thigh Tissue | 0.046 | -0.084 | 0.109 | -0.031 | -0.203 | -0.226 | -0.108 | -0.232 |
|  | 0.740 | 0.527 | 0.397 | 0.808 | 0.110 | 0.075 | 0.401 | 0.067 |
| T3 Total Thigh Tissue | 0.256 | 0.210 | 0.252 | 0.082 | 0.014 | 0.085 | -0.157 | -0.182 |
|  | 0.057 | 0.107 | 0.045 | 0.519 | 0.914 | 0.503 | 0.215 | 0.149 |
| T2 Thigh Muscle Mass | -0.042 | -0.079 | 0.105 | -0.036 | -0.242 | -0.233 | -0.091 | -0.153 |
|  | 0.759 | 0.552 | 0.413 | 0.779 | 0.056 | 0.067 | 0.476 | 0.230 |
| T3 Thigh Muscle Mass | 0.226 | 0.213 | 0.291 | 0.103 | -0.032 | 0.036 | -0.261 | -0.277 |
|  | 0.094 | 0.102 | 0.020 | 0.416 | 0.803 | 0.777 | 0.038 | 0.027 |
| T2 Subcutaneous Thigh Fat | 0.146 | -0.054 | 0.096 | -0.022 | -0.134 | -0.187 | -0.105 | -0.289 |
|  | 0.289 | 0.682 | 0.455 | 0.865 | 0.294 | 0.143 | 0.414 | 0.021 |
| T3 Subcutaneous Thigh Fat | 0.139 | 0.186 | 0.212 | 0.116 | 0.036 | 0.082 | -0.129 | -0.144 |
|  | 0.307 | 0.154 | 0.093 | 0.363 | 0.775 | 0.520 | 0.309 | 0.257 |
| T2 Total Arm Tissue | 0.137 | -0.033 | -0.002 | -0.046 | -0.186 | -0.228 | -0.156 | -0.210 |
|  | 0.318 | 0.802 | 0.990 | 0.720 | 0.144 | 0.073 | 0.222 | 0.098 |
| T3 Total Arm Tissue | 0.183 | 0.050 | 0.158 | 0.100 | 0.011 | 0.008 | -0.212 | -0.194 |
|  | 0.176 | 0.702 | 0.213 | 0.431 | 0.934 | 0.947 | 0.093 | 0.125 |
| T2 Arm Muscle Mass | 0.131 | -0.040 | 0.051 | -0.016 | -0.162 | -0.194 | -0.039 | -0.101 |
|  | 0.339 | 0.765 | 0.694 | 0.903 | 0.205 | 0.127 | 0.762 | 0.433 |
| T3 Arm Muscle Mass | 0.258 | 0.115 | 0.145 | 0.097 | -0.051 | -0.028 | -0.220 | -0.226 |
|  | 0.055 | 0.383 | 0.255 | 0.444 | 0.691 | 0.827 | 0.080 | 0.072 |
| T2 Subcutaneous Arm Fat | 0.083 | -0.051 | -0.013 | -0.059 | -0.201 | -0.245 | -0.236 | -0.309 |
|  | 0.549 | 0.700 | 0.919 | 0.647 | 0.114 | 0.053 | 0.062 | 0.014 |
| T3 Subcutaneous Arm Fat | 0.080 | -0.044 | 0.129 | 0.093 | 0.081 | 0.055 | -0.157 | -0.134 |
|  | 0.557 | 0.736 | 0.310 | 0.464 | 0.526 | 0.668 | 0.215 | 0.290 |
| T2 Length | 0.034 | -0.120 | 0.103 | -0.014 | -0.213 | -0.229 | -0.176 | -0.285 |
|  | 0.807 | 0.364 | 0.424 | 0.913 | 0.094 | 0.072 | 0.168 | 0.024 |
| T3 Length | 0.080 | -0.025 | 0.124 | 0.082 | -0.155 | -0.135 | -0.320 | -0.368 |
|  | 0.557 | 0.850 | 0.334 | 0.522 | 0.226 | 0.291 | 0.011 | 0.003 |
| T4 Length | 0.071 | 0.031 | 0.237 | 0.177 | 0.064 | 0.103 | 0.037 | 0.024 |
|  | 0.602 | 0.815 | 0.059 | 0.162 | 0.613 | 0.419 | 0.774 | 0.848 |
| T4 BMI (kg/m^2^) | 0.012 | 0.146 | 0.012 | -0.108 | -0.075 | 0.002 | 0.089 | 0.164 |
|  | 0.927 | 0.266 | 0.927 | 0.396 | 0.558 | 0.985 | 0.482 | 0.196 |
| T4 Head Circumference | 0.178 | 0.308 | 0.382 | 0.228 | 0.047 | 0.070 | -0.001 | 0.098 |
|  | 0.216 | 0.028 | 0.005 | 0.101 | 0.741 | 0.620 | 0.994 | 0.484 |
| T4 Thoracic Circumference | 0.030 | 0.146 | 0.330 | 0.100 | -0.002 | 0.066 | 0.103 | 0.050 |
|  | 0.835 | 0.307 | 0.016 | 0.478 | 0.990 | 0.641 | 0.462 | 0.723 |
| T4 Abdominal Circumference | 0.010 | 0.135 | 0.193 | 0.073 | 0.077 | 0.057 | -0.009 | -0.054 |
|  | 0.944 | 0.346 | 0.166 | 0.605 | 0.585 | 0.683 | 0.949 | 0.703 |
| T4 Weight | 0.051 | 0.185 | 0.229 | 0.010 | 0.022 | 0.065 | 0.122 | 0.059 |
|  | 0.727 | 0.197 | 0.102 | 0.944 | 0.877 | 0.648 | 0.390 | 0.676 |
| T2 Fetal Weight | 0.073 | -0.135 | 0.022 | -0.094 | -0.152 | -0.191 | -0.177 | -0.314 |

Spearman’s test. SCFT: abdominal subcutaneous fat thickness; and BMI: Body Mass Index. Time 1 (T1) = gestational age ≤ 15 weeks; time 2 (T2) = gestational age 20-26 weeks; time 3 (T3) = gestational age 30-36 weeks; and time 4 (T4) = at delivery.

| **Table S2(B)**. Correlation of DNA methylation itself with gene expression in maternal blood, cord blood, maternal decidua and placental villi tissues. | | | | | | | | |
| --- | --- | --- | --- | --- | --- | --- | --- | --- |
|  | Maternal Blood *H19DMR*  Methylation | Cord  Blood  *H19DMR*  Methylation | Placental  Villi  *H19DMR* Methylation | Maternal Decidua *H19DMR* Methylation | Placental Villi  *H19*  Expression | Placental Villi  *IGF2*  Expression | Maternal Decidua  *H19* Expression | Maternal Decidua  *IGF2* Expression |
| Maternal Blood *H19DMR* Methylation | - | 0.331 | 0.095 | 0.236 | -0.027 | -0.093 | -0.239 | -0.328 |
|  | - | 0.013 | 0.488 | 0.080 | 0.846 | 0.497 | 0.077 | 0.014 |
| Cord Blood *H19DMR* Methylation | 0.331 | - | 0.381 | 0.348 | 0.125 | 0.142 | 0.071 | -0.010 |
|  | 0.013 | - | 0.003 | 0.006 | 0.340 | 0.279 | 0.589 | 0.942 |
| Placental Villi *H19DMR* Methylation | 0.095 | 0.381 | - | **0.788** | 0.022 | 0.047 | -0.073 | -0.057 |
|  | 0.488 | 0.003 | - | 0.000 | 0.862 | 0.709 | 0.569 | 0.657 |
| Maternal Decidua *H19DMR* Methylation | 0.236 | 0.348 | 0.788 | - | -0.080 | -0.138 | -0.120 | -0.079 |
|  | 0.080 | 0.006 | 0.000 | - | 0.528 | 0.278 | 0.345 | 0.533 |
| Placental Villi *H19* Expression | -0.027 | 0.125 | 0.022 | -0.080 | - | **0.912** | 0.386 | 0.281 |
|  | 0.846 | 0.340 | 0.862 | 0.528 | - | 0.000 | 0.002 | 0.025 |
| Placental Villi *IGF2* Expression | -0.093 | 0.142 | 0.047 | -0.138 | 0.912 | - | 0.406 | 0.350 |
|  | 0.497 | 0.279 | 0.709 | 0.278 | 0.000 | - | 0.001 | 0.005 |
| Maternal Decidua *H19* Expression | -0.239 | 0.071 | -0.073 | -0.120 | 0.386 | 0.406 | - | **0.841** |
|  | 0.077 | 0.589 | 0.569 | 0.345 | 0.002 | 0.001 | - | 0.000 |
| Maternal Decidua *IGF2* Expression | -0.328 | -0.010 | -0.057 | -0.079 | 0.281 | 0.350 | 0.841 | - |
|  | 0.014 | 0.942 | 0.657 | 0.533 | 0.025 | 0.005 | 0.000 | - |

Spearman’s test.
